# Respective roles of migration and social deprivation for virological non-suppression in HIV-infected adults on antiretroviral therapy in France

**DOI:** 10.1371/journal.pone.0213019

**Published:** 2019-03-07

**Authors:** Mariem Raho-Moussa, Marguerite Guiguet, Céline Michaud, Patricia Honoré, Christia Palacios, François Boué, Mohammed Azghay, Imad Kansau, Véronique Chambrin, Tania Kandel, Marion Favier, Elsa Miekoutima, Naomi Sayre, Carole Pignon, Michka Shoai, Olivier Bouchaud, Sophie Abgrall

**Affiliations:** 1 AP-HP, Hôpital Antoine Béclère, Service de Médecine interne/Immunologie clinique, Clamart, France; 2 Sorbonne Universités, UPMC Université Paris 06, INSERM, Institut Pierre Louis d’épidémiologie et de Santé Publique (IPLESP UMRS 1136), Paris, France; 3 AP-HP, Hôpital Avicenne, Service de Maladies Infectieuses et Tropicales, Bobigny, France; 4 Université Paris-Saclay, Univ. Paris-Sud, UVSQ, Le Kremlin-Bicêtre, France; 5 Université Paris 13, Bobigny, France; 6 CESP INSERM U1018, Le Kremlin-Bicêtre, France; Medical University of Warsaw, POLAND

## Abstract

Barriers to achieve sustained HIV virological suppression on antiretroviral therapy (ART) jeopardize the success of the 90:90:90 UNAIDS initiative which aims to end the HIV/AIDS epidemic. In France, where access to ART is free and universally available, we analyze the way in which social determinants of health (i.e. cultural, environmental) and economic factors might influence virological outcomes. A cross-sectional study was performed in two hospitals located in Paris area. All consecutive people living with HIV (PLHIV) on ART for at least 6 months attending the outpatient clinics between 01/05/2013 and 31/10/2014 answered an individual score of deprivation, EPICES, retrieving information on health insurance status, economic status, family support and leisure activity. This score varies from 0 to 100 with deprivation state defined above 30.17. Factors associated with HIV viral load >50 copies/ml were assessed by logistic regression modeling with a backward stepwise selection to select the final multivariable model. Sensitivity analyses were performed using two other thresholds for virological non-suppression (100 or 200 copies/ml). Overall, 475 PLHIV were included (53% male, median age 47 years, 66% not born in France mainly in a sub-Saharan African country). Half of French natives and 85% of migrants were classified as deprived. Median duration on ART was 9.7 years with virological suppression in 95.2% of non-deprived participants and in 83.5% of deprived ones (p = 0.001). The final multivariable model retained ART tiredness, younger age, a previous AIDS event and social deprivation (adjusted Odds Ratio, 2.9; 95%CI, 1.2–7.0) as determinants of virological non-suppression but not migration in itself. When using separate components of EPICES score, reporting economic difficulties and non-homeownership were associated with virological non-suppression. In addition to interventions focusing on cultural aspects of migration, social interventions are needed to help people with social vulnerability to obtain sustained responses on ART.

## Introduction

Clinical prognosis and survival among people living with HIV (PLHIV) on antiretroviral therapy (ART) depend on sustained virological suppression and CD4 cell count recovery. However, social determinants of health (ie cultural, environmental) and economic factors might influence outcomes after ART initiation. In Europe, poorer virological response on ART has been observed in migrants from sub-Saharan Africa (SSA) compared to non-migrant people [1–3]. In France, one study reported a higher risk of virological failure among heterosexual migrants mainly from SSA, in comparison with men having sex with men (MSM) [4], whereas other studies did not observe any differences between these two groups [5], or reported a higher risk of failure among non-homosexual men, whatever their geographic origin [6]. Whether this can be explained by different socioeconomic position, education or lifestyle is still questionable [7,8].

In the French context of free universal access to care, we aimed to assess association between migration, socioeconomic status and virological suppression in PLHIV on ART in two hospitals located in Paris area.

## Methods

### Participants

We conducted a cross-sectional study in HIV-infected adults (≥ 18 years) treated with ART for at least 6 months attending the outpatient clinic for their one-day annual medical evaluation in two French university clinical centers located in Seine-Saint Denis department in the North of Paris (hospital 1) and in the Haut-de-Seine department in the South of Paris (hospital 2). In these centers, nearly all PLHIV in care can speak French, otherwise health care providers are used to call Interservice Migrant (ISM), a professional service for telephone translation. Between 01/05/2013 and 31/10/2014, all consecutive patients were included. Patients’ medical data were retrieved from the Nadis and from the DOMEVIH electronic medical records. According to the French legislation, both records received approval from the French “Comité consultatif sur le traitement de l’information en matière de recherche dans le domaine de la santé” (Registration number 15.196 and JO 17.01.1992 respectively). All the participants were informed of the study and gave their written informed consent to allow the use of their personal clinical data. The two university clinical centers are part of Assistance Publique-Hopitaux de Paris (AP-HP) that allowed the use of administrative, social and clinical data for non-interventional research to improve quality of care (https://www.aphp.fr/protection-des-donnees-personnelles).

### Data collection

Demographic, clinical, therapeutic and biological data were retrieved from the medical record. All participants answered an individual score of deprivation, the *Evaluation de la précarité et des inégalités de santé dans les centres d’examens de santé* (EPICES; Evaluation of the Deprivation and Inequalities of Health in Healthcare Centres), that takes into account the multiple dimensions of socioeconomic conditions including psychological, social and economic aspects [9,10]. The EPICES score is based on the responses to 11 binary questions (marital status, health insurance status, economic status, family support and leisure activity). It varies from 0 (the least deprived) to 100 (the most deprived) [9,10] with a deprivation state defined as a score ≥ 30.17, a threshold established in a large cohort study carried out by the Centre technique d’appui et de formation des centres d’examens de santé (CETAF; Technical Centre of Support and Training for Health Centres) [9]. A short questionnaire was completed on social variables not included in the EPICES score, such as education, occupation, disclosure, as well as self-perceived treatment efficacy and health (S1 Table). In particular, self-perceived ART effectiveness and self-perceived ART toxicity were asked for with four levels of answer (no efficiency / low efficiency / high efficiency / very high efficiency; no toxicity / low toxicity / high toxicity / very high toxicity). ART tiredness was looked for through the following assessment “With regard to the taking of ART, would you say to yourself that you are: not at all tired / a little tired / tired / very tired” and the last two levels were grouped to indicate ART tiredness.

### Statistical analyses

Descriptive statistics are shown as median and interquartile ranges (IQR) or numbers and percentages with the comparisons based on the Kruskal-Wallis test for continuous variables and X2 tests for categorical variables. Factors associated with virological non-suppression, defined as plasma viral load (VL) > 50 copies/ml, at the date of the study outpatient visit were assessed by using logistic regression models. The selection of covariates was based on a backward stepwise technique with a step-by-step exclusion of a single variable. Eight variables were associated with virological non-suppression with a p value less than 0.10 in the univariable analysis (age, sex-transmission risk group, country of birth, EPICES score, perceived ART effectiveness, ART tiredness, ART regimen, previous AIDS-defining event) and were investigated, except for perceived ART effectiveness and ART regimen that could have been modified by viral replication. Starting from the full model with all variables, Akaike Information Criterion (AIC) for the model and Type III p-values for each variable were calculated. At each step, the variable with the highest p-value was dropped and the reduced model was fitted until a model including intercept only. The final model was the model with the lowest AIC and consequently the best goodness-of-fit. Additional multivariable analysis was also performed without selection of variables. Two multivariable analyses were separately investigated with deprivation defined either using cut-off at proposed EPICES score threshold (model 1) or using individual components of EPICES score (model 2). Finally, self-reporting ART tiredness was omitted from potential explanatory variables in order to compare estimates associated with social deprivation in the models adjusting or not adjusting on ART tiredness. In sensitivity analyses, we used two other thresholds to define virological non-suppression, 100 copies/ml or 200 copies/ml. The results of logistic regression modelling were presented as adjusted odds ratios (aORs) and 95% confidence intervals (CIs). The statistical analyses were performed with SAS v9.4 software (SAS Institute Inc.).

## Results

A total of 475 patients on ART were included (53% male, median age 47 years, median duration of HIV infection 11.5 years) of whom 349 from hospital 1 and 126 from hospital 2. Overall, 315 (66%) participants were not born in France, of whom 231 (73%) were born in SSA, 18 (6%) in an European country, and 66 (21%) in another foreign country. All PLHIV have been on ART for at least 6 months; median duration on ART was 9.7 years (IQR, 4.5–16.2) with only 18 (3.8%) individuals on ART for less than one year. Patients’ characteristics according to the hospital are described in Table 1. More patients were born in a country from SSA in hospital 1 while more patients were men who have sex with men (MSM) or have been infected through intravenous drug use (IDU) in hospital 2. Among the whole population, median (IQR) EPICES score was 47.9 (29.0–63.9) and 74% could be classified as deprived. Deprivation was more frequent in hospital 1 (77%) than in hospital 2 (63%) (p = 0.003), in women (84%) and in non MSM men (73%) than in MSM (43%) (p<0.0001), and more frequent in migrants from SSA (87%) or from other foreign country (81%) than in PLHIV born in France (51%) (p<0.0001). Compared to non-deprived patients, deprived patients were slightly younger (median age, 47 vs 50 years, p = 0.01), less educated (39% with educational level lower than secondary school vs 17%, p<0.0001) and less employed (52% employed vs 68%, p = 0.003). There were no differences in duration of HIV infection, previous AIDS, CD4 nadir, ART duration or ART combination.

**Table 1:** Characteristics of 475 HIV-infected adults on antiretroviral therapy included in two tertiary hospital located in suburbs of Paris area, France.

	All	Hospital 1	Hospital 2	p	Deprived^a^	Not deprived	P	Detectable HIV VL^b^	Undetectable HIV VL	p
Total	475 (100.0)	349 (100.0)	126 (100.0)		351 (100.0)	124 (100.0)		64 (100.0)	411 (100.0)	
Age, years	47 (41–54)	48 (42–54)	46 (38–52)	0.009	47 (41–53)	50 (43–56)	0.01	46 (38–52)	47 (41–54)	0.09
Sex, men	251 (52.8)	187 (53.6)	64 (50.8)	0.60	164 (46.7)	87 (70.2)	<0.0001	27 (42.2)	224 (54.5)	0.08
Transmission risk group				0.0002			<0.0001			0.03
MSM	53 (11.2)	28 (8.0)	25 (19.8)		23 (6.6)	30 (24.2)		1 (1.6)	52 (12.7)	
IDU	44 (9.2)	28 (8.0)	16 (12.7)		28 (8.0)	16 (12.9)		5 (7.8)	39 (9.5)	
Heterosexual men	166 (35.0)	136 (39.0)	30 (23.8)		121 (34.5)	45 (36.3)		21 (32.8)	145 (35.3)	
Heterosexual women	212 (44.6)	157 (45.0)	55 (43.7)		179 (51.0)	33 (26.6)		37 (57.8)	175 (42.6)	
Country of birth				<0.0001			<0.0001			0.03
France	160 (33.7)	96 (27.5)	64 (50.8)		82 (23.4)	78 (62.9)		13 (20.3)	147 (35.8)	
Sub-Saharan Africa	231 (48.6)	188 (53.9)	43 (34.1)		201 (57.3)	30 (24.2)		35 (54.7)	196 (47.7)	
Other	84 (17.7)	65 (18.6)	19 (15.1)		68 (19.4)	16 (12.9)		16 (25.0)	68 (16.6)	
Living with children	187 (39.9)	125 (35.8)	62 (51.7)	0.002	140 (40.3)	47 (38.5)	0.75	27 (43.6)	160 (39.3)	0.58
Educational level < secondary school	156 (33.0)	138 (39.7)	18 (14.4)	<0.0001	135 (38.7)	21 (16.9)	<0.0001	22 (34.9)	134 (32.7)	0.77
Employment (formal or informal, full or part-time)	268 (56.4)	186 (53.3)	82 (65.1)	0.03	184 (52.4)	84 (67.7)	0.003	32 (50.0)	236 (57.4)	0.28
EPICES score	47.9 (29.0–63.9)	48.5 (31.4–65.1)	44.4 (21.9–62.1)	0.02	55.6 (45.0–69.2)	16.0 (8.3–23.1)	<0.0001	55.9 (41.7–71.6)	46.2 (26.6–63.9)	0.0006
Deprivation, Epices score≥30.17	351 (73.9)	271 (77.6)	80 (63.5)	0.003				58 (90.6)	293 (71.3)	0.0007
BMI	25.5 (22.5–29.3)	25.8 (22.7–29.5)	24.5 (21.3–27.8)	0.01	26.0 (22.4–29.7)	24.5 (22.6–27.5)	0.04	25.6 (22.1–29.9)	25.5 (22.5–29.1)	0.66
HIV status disclosure	351 (75.2)	260 (75.1)	91 (75.2)	1.00	24.5 (71.0)	106 (86.9)	0.0004	43 (68.2)	308 (76.2)	0.21
Perceived ART effectiveness	451 (97.4)	334 (97.7)	117 (96.7)	0.52	332 (97.4)	119 (97.5)	1.00	53 (89.8)	398 (98.5)	0.002
Perceived ART toxicity	96 (21.5)	59 (17.6)	37 (33.3)	0.0008	60 (18.3)	36 (30.2)	0.009	11 (20.7)	85 (21.6)	1.00
ART tiredness	104 (22.7)	72 (21.2)	32 (27.1)	0.20	88 (26.3)	16 (13.0)	0.002	21 (37.5)	83 (20.8)	0.01
Duration of HIV infection, years	11.5 (6.9–18.7)	11.5 (6.5–17.8)	12.0 (7.7–20.0)	0.14	11.4 (6.7–17.9)	12.2 (7.4–20.0)	0.16	11.4 (6.8–16.7)	11.5 (6.9–18.8)	0.57
Duration on ART, years	9.7 (4.5–16.2)	9.7 (4.1–15.8)	9.9 (5.3–17.6)	0.06	9.7 (4.4–15.6)	9.4 (5.0–17.4)	0.27	9.5 (5.0–15.3)	9.7 (4.5–16.5)	0.66
Year starting ART				0.08			0.07			0.77
Before 2000	146 (30.7)	100 (28.6)	46 (36.5)		99 (28.2)	47 (37.9)		18 (28.1)	128 (31.1)	
2000–2004	81 (17.1)	64 (18.3)	17 (13.5)		68 (19.4)	13 (10.5)		11 (17.2)	70 (17.0)	
2005–2009	124 (26.1)	86 (24.6)	38 (30.2)		91 (25.9)	33 (26.6)		20 (31.2)	104 (25.3)	
2010 and after	124 (26.1)	99 (28.4)	25 (19.8)		93 (26.5)	31 (25.0)		15 (23.4)	109 (26.5)	
Antiretroviral combination				<0.0001			0.67			0.0006
2NRTI + 1 PI	171 (36.0)	109 (31.2)	62 (49.2)		130 (37.0)	41 (33.1)		31 (48.4)	140 (34.1)	
2NRTI+ 1 NNRTI	185 (39.0)	159 (45.6)	26 (20.6)		133 (37.9)	52 (41.9)		11 (17.2)	174 (42.3)	
Other	119 (25.0)	81 (23.2)	38 (30.2)		88 (25.1)	31 (25.0)		22 (34.4)	97 (23.6)	
Previous AIDS-defining event	155 (32.7)	127 (36.4)	28 (22.4)	0.004	122 (34.8)	33 (26.8)	0.12	28 (43.7)	127 (31.0)	0.05
Nadir CD4 (/mm^3^)	220 (90–340)	214 (86–339)	230 (125–347)	0.17	217 (90–334)	234 (100–376)	0.39	193 (100–300)	226 (90–350)	0.23
Zenith HIV VL (log_10_ copies/ml)	4.63 (3.70–5.20)	4.70 (3.79–5.28)	4.41 (3.43–5.08)	0.009	4.57 (3.70–5.19)	4.82 (3.72–5.22)	0.46	4.82 (3.62–5.31)	4.58 (3.69–5.19)	0.41
CD4 (/mm^3^)	544 (376–734)	530 (369–734)	572 (394–732)	0.44	513 (352–701)	602 (466–796)	0.0005	363 (188–488)	579 (412–760)	<0.0001
CD4/CD8	0.78 (0.45–1.11)	0.74 (0.45–1.07)	0.81 (0.51–1.21)	0.08	0.73 (0.42–1.07)	0.84 (0.55–1.21)	0.005	0.38 (0.24–0.74)	0.83 (0.53–1.17)	<0.0001
HIV VL < = 50 copies/ml	411 (86.5)	302 (86.5)	109 (86.5)	1.00	293 (83.5)	118 (95.2)	0.001			

Data are median (Q1-Q3) or n (%)

^a^ Deprived defined by Epices score≥30.17

^b^ Detectable HIV Viral Load defined by HIV viral load >50 copies/ml

Missing data: living with children (n = 6); educational level (n = 2); BMI (n = 1); HIV disclosure (n = 8); perceived CART effectiveness (n = 12); perceived CART toxicity (n = 28); CART tiredness (n = 12); previous AIDS-defining event (n = 1); nadir CD4 cell count (n = 10); CD4/CD8 ratio (n = 6)

Analysis of the eleven components of EPICES score showed that one over four respondents had previously been in contact with social workers while reporting financial difficulties in the previous month was reported by half of respondents (Table 2).

**Table 2:** Components of EPICES score reported by 475 HIV-infected adults on antiretroviral therapy included in two tertiary hospitals located in suburbs of Paris area, France.

	N (%)
*Total*	475 (100.0%)
Help from social worker	125 (26.3%)
No full health insurance	168 (35.4%)
Living alone	266 (56.0%)
No owner of his/her housing	395 (83.2%)
Financial difficulties in the month	276 (58.1%)
No sport activity in the previous year	274 (57.7%)
No outing to shows in the previous year	288 (60.6%)
No holiday away from home in the previous year	204 (42.9%)
No relation with relatives within the last six months	67 (14.1%)
No possible hosting in the social network if necessary	177 (37.3%)
No possible financial help in the social network if necessary	233 (49.1%)

Virological non-suppression >50 copies/ml was observed in 64 (13.5%) patients (Table 1), more frequently in migrants from SSA (35/231, 15.1%) or from other foreign country (16/84, 19.1%) than in PLHIV born in France (13/160, 8.1%) (p = 0.03) and more frequently in deprived people (58/351, 16.5%) than in non-deprived ones (6/124, 4.8%) (p = 0.0007). The final multivariable model retained four variables associated with the risk of virological non-suppression among which social deprivation but not sex-transmission group or country of birth. As shown in Table 3, deprivation defined by EPICES score was associated with an increased risk of virological non-suppression (aOR, 2.9; 95%CI, 1.2 to 7.0). When assessing separate components of EPICES score, non-homeownership (aOR, 9.0; 95%CI, 1.2 to 67.5) and reporting economic difficulties (aOR, 2.9; 95%CI, 1.4 to 6.1) were associated with virological non-suppression. In both final models, younger age and previous AIDS events were also risk factors for virological non-suppression. People reporting ART tiredness were twice more likely to not have virological suppression (aOR, 2.2; 95%CI, 1.2 to 4.1). Additional analysis omitting self-reporting ART tiredness did not modify the selection of retained variables in the multivariable model with unsubstantial changes in estimates, in particular for the risk associated with EPICES score above the threshold of social deprivation (aOR, 3.1; 95% CI, 1.3 to 7.6).

**Table 3:** Factors associated with a detectable HIV viral load (VL>50 copies/ml) in 475 HIV-infected adults on antiretroviral therapy included in two tertiary hospital located in suburbs of Paris area, France. Two multivariable analyses were separately investigated with deprivation defined either using cut-off at proposed EPICES score threshold (model 1) or using individual components of EPICES score (model 2).

		Model 1 aOR (95%CI)	Model 2 aOR (95%CI)
Age	<35 yrs	3.60 (1.67–7.76)	3.44 (1.57–7.56)
	35+	1.00	1.00
ART tiredness	Yes	2.23 (1.21–4.12)	2.15 (1.14–4.03)
	No	1.00	1.00
Previous AIDS-defining event	Yes	2.01 (1.11–3.63)	2.30 (1.26–4.21)
	No	1.00	1.00
Deprivation	Yes, Epices score≥30.17	2.89 (1.18–7.04)	
	No	1.00	
*Components of EPICES score*:			
Owner of his/her housing	Yes		1.00
	No		8.96 (1.19–67.46)
Financial difficulties in the previous month	No		1.00
	Yes		2.90 (1.38–6.10)

aOR, adjusted odds ratio.

In supplementary analysis that also included country of birth and sex-transmission risk group, the pejorative effect of social deprivation was still observed (aOR, 2.4; 95%CI, 0.96–6.2) while sex-transmission risk group (p = 0.49) and country of birth (p = 0.88) were not associated with virological non-suppression. Sensitivity analyses with other thresholds for virological non-suppression definition (9.5%, and 7.2%, of patients with HIV RNA levels>100 or 200 copies/ml respectively) showed similar results (data not shown).

## Discussion

In this cross-sectional study performed in a high income country with universal free access to HIV care and ART, deprivation was a major determinant of virological non-suppression on long term ART with VL>50 copies/ml in 17% of deprived patients and 5% of non-deprived patients. When using separate components of the EPICES score, reporting economic difficulties was associated with virological non-suppression. Other adjusted risk factors were reporting ART tiredness, younger age, and a previous AIDS event, but not migration in itself.

Deprivation has been proposed to enlarge the notion of poverty and the EPICES score has been developed to measure deprivation at the individual level. This score is reliable when compared to the Townsend and the Carstairs indices and has been identified as an independent determinant of worse health status and premature mortality [10]. In different previous French studies, 58% of HIV-infected adults were classified as deprived using the EPICES score [11], 49% reported financial difficulties [12], 30% reported material deprivation [13]. The impressive level of deprivation in our study with nearly three over four participants classified as deprived using the EPICES score could be linked to the location of hospitals in two suburbs near Paris. This high level of deprivation has been also reported among HIV-infected patients from the North Paris area [14]. Sociodemographic disparities in health have been challenged towards cultural aspects with recruitment of health navigators in some infectious diseases departments. Involvement of social workers ought also to be reinforced in order to decrease health inequalities.

The respective role of sociodemographic characteristics and economic factors on response to ART cannot be easily distinguished. In absence of data on socioeconomic status, geographical origin, sex and HIV transmission group have been shown to modify outcome after ART initiation, partly because of later ART initiation in migrant men than in non-migrants [15,16,17]. In various contexts, migrants have been reported to have poorer outcomes with higher risk of virological failure [1,2,6] possibly due to lower rates of adherence or retention in care [18,19]. In our multivariable analyses, neither HIV transmission group nor migration were associated with virological non-suppression after adjusting for deprivation highlighting the role of social disadvantage rather than geographic origin or HIV transmission group per se, in agreement with analyses of educational attainment, a proxy for socioeconomic status, that remained associated with inequalities of outcomes on ART when adjusted for potential confounders and particularly for HIV transmission category [20]. In a recent study also performed in another country with universal free access to health care, in which information on both migration and socioeconomic status were available, socioeconomic disadvantages, particularly financial difficulties, were stronger determinants of virological non-suppression than migration status [21].

In the French ANRS-VESPA 2 study, relationship has been observed between virological response and both education and employment status while association with material deprivation was no more significant after adjustment [13]. On the other hand, when using components of EPICES score in our study, main risk factors for virological non-suppression were financial and housing status but not education or employment. These apparent conflicting results could be explained by underlying mechanisms for the negative effect of lower socioeconomic status on ART outcomes. The role of non-adherence has been suggested as a mediator in the relationship between socioeconomic status and virological response [19]. In a recent French study of women on ART, reporting financial difficulties decreased the likelihood of coping with common side effects and was associated with changes to the ART regimen without prior discussion with the healthcare provider [12]. It is noteworthy that people reporting ART tiredness in our study were twice more likely to have detectable VL. Even if ART tiredness was more frequently reported by PLHIV with social deprivation, poor socioeconomic characteristics cannot simply be equated with this subjective self-report perception as indicated by the close estimates associated with social deprivation in the models adjusting or not adjusting on ART tiredness. On the other hand, our results pointed out the importance of long-term health-provider support for all PLHIV. Positive message such as the U = U (undetectable = untransmissable) slogan might help motivating pursuit of ART and attainment of stable virological suppression [22].

The strength of the study was the inclusion of PLHIV on care in two hospitals located in two suburb areas with more patients from SSA in one hospital and more patients MSM or infected through IDU in the other. The main limitation was the cross-sectional design which limits the interpretation of our results. Second, we used EPICES score as a measure of social deprivation while this score has not been specifically validated in migrant population. In particular, sociodemographic differences between the two hospitals translated into some components of the EPICES score such as home ownership more frequently reported by PLHIV born in France, but the strong penalty of financial difficulties was similar in the two hospitals. Third, information were missing that could have offered insights for our results. Migration was only defined by country of birth without differentiating according to the duration of stay in France. Self-perception of ART tiredness was asked for without specific measurement of ART adherence or retention in care in the previous months. Concerning our outcome, virological suppression was coded based on a single VL which could overestimate the percent of PLHIV with stable virological suppression, ultimate goal of ART [23]. Virological non-suppression was defined as virological replication using different thresholds of 50, 100, or 200 copies/ml. However, even low-level viremia has been shown to be associated with increased risk of antiretroviral drug resistance and virological failure [24].

## Conclusions

In our study that included PLHIV who had been on ART for a median duration of nearly 10 years, HIV viral load was undetectable (<50 copies/ml) in 86.5% of them at the time of the survey close to the challenge of the final UNAIDS 90-90-90 targets (i.e. 90% of HIV-infected people diagnosed, 90% of HIV-positive people on ART, 90% of people on ART with suppressed VL) which aims to end the HIV/AIDS epidemic. However, in a context of free universal access to care, deprivation and especially reporting financial difficulties are still risk factors for virological non-suppression. All barriers preventing long-term uninterrupted suppressed HIV VL should be identified and considered by health-care providers. In addition to interventions focusing on cultural aspects of migration aimed at increasing HIV diagnosis and linkage to care in specific HIV infected key population, social interventions are needed to help people with social vulnerability to obtain sustained responses on ART.

## Supporting information

S1 Table Study questionnaire. (DOCX)

S1 File The dataset used for the analysis. (XLSX)
