# Supplementary material for: Respective roles of migration and social deprivation for virological non-suppression in HIV-infected adults on antiretroviral therapy in France
Source: PLoS One. 2019 Mar 7;14(3):e0213019. doi: 10.1371/journal.pone.0213019 (PMC6405133; doi:10.1371/journal.pone.0213019)
Supplement: S1 Table — (DOCX) [file pone.0213019.s001.docx]

**Study questionnaire**

| **Date** | | **Anonymity : LNFNYY** | |
| --- | --- | --- | --- |
|  |  | | **Year of birth :** |

SEX ❑ M ❑ F

1. Country of birth: ❑ France ❑ Other

1. If other: which country of birth : ……………………….

If other:

French nationality acquired ❑ YES ❑ NO

Residence permit with work permit ❑ YES ❑ NO

Residence permit without work permit ❑ YES ❑ NO

Illegal status ❑ YES ❑ NO

1. Currently,

**❑** You cannot read or write (French)

**❑** You can read or you can write (French)

**❑** You can read and you can write (French)

1. What is your higher education degree ?

**❑** None

**❑** End of primary school

**❑** End of lower secondary school

**❑** End of upper secondary school

**❑** Tertiary school

**❑** Other (specify) :…………………………………………

1. Your current professional activity is:

**❑** Salaried or self-employed activity

**❑** Full-time activity (≥ 35h/week) **❑** or part-time

**❑** Solidarity labor income

**❑** Disability income

**❑** Unemployment benefit

**❑** Other social assistance (help for housing…)

**❑** Retired

**❑** Without income

**EPICES SCORE**

1. **Do you sometimes ask for help from a social worker? ❑** YES **❑** NO
2. **Do you have a full health insurance? ❑** YES **❑** NO
3. Social insurance:

**❑ **General social security system

**❑ **Additional private health insurance

**❑ **Universal Health Cover

**❑ ** Universal Complementary Health Cover

**❑ **100% social coverage for long duration disease

**❑ **State medical assistance

**❑ **Free medical centre

1. **Do you live as a couple? ❑** YES **❑** NO
2. Number of dependent children (living in the dwelling):

1. **Do you own your home? ❑** YES **❑** NO
2. If we consider your current home:

**❑** You own your home in France

**❑** You rent your home in France

**❑** You live at a friend's house

**❑** You live with your parents or with other family members

**❑** You live in a hostel, a worker's home or a residential centre

**❑** Other:…………………………………………………………………….

1. Would you say that your current home is:

**❑** Not at all comfortable

**❑** Uncomfortable

**❑** Quite comfortable

**❑** Very comfortable

1. **Are there periods in the month when you have real financial difficulties to meet your needs (food, rent, electricity ...)? ❑** YES **❑** NO
2. **Have you ever had sports in the last 12 months? ❑** YES **❑** NO
3. **Have you been to a show in the last 12 months? ❑** YES **❑** NO
4. **Hae you gone on vacation in the last 12 months? ❑** YES **❑** NO
5. **Have you had any contact with family members other than your parents and/or children in the last 6 months? ❑** YES **❑** NO
6. **In case of difficulty, are there people around you that you can count on to accommodate you for a few days in case of need? ❑** YES **❑** NO
7. **In case of difficulty, are there people around you that you can count on to provide you with material financial assistance? ❑** YES **❑** NO

**OTHER**

1. Smoking: do you smoke everyday? **❑** YES **❑** NO
2. Alcohol > 30 g/d (3 glasses of wine or 3 beers) **❑** YES **❑** NO
3. Drug use (crack/cocaine/cannabis/héroin...): **❑** YES **❑** NO
4. Have you talk about your HIV infection to at least one person around you (relative, friend, neighbor, professional relationship...): **❑** YES **❑** NO
5. According to you, the treatment you are currently taking has:

**❑** Very high efficiency

**❑** High efficiency

**❑** Low efficiency

**❑** No efficiency

1. According to you, the treatment you are currently taking has:

**❑** Very high toxicity

**❑** High toxicity

**❑** Low toxicity

**❑** No toxicity

1. With regard to the taking of ART, would you say to yourself that you are:

**❑** Very tired

**❑** Tired

**❑** A little tired

**❑** Not at all tired
